# Human challenge models for vaccine development—strengths, limitations, and expansion into endemic settings: a HIC-Vac meeting report

**DOI:** 10.1093/immadv/ltaf004

**Published:** 2025-02-08

**Authors:** Helen R Wagstaffe, Stephanie Ascough, Peter J M Openshaw, HIC-Vac meeting contributors

**Affiliations:** Department of Infectious Diseases, Imperial College London, London, United Kingdom; Department of Infectious Diseases, Imperial College London, London, United Kingdom; National Heart and Lung Institute, Imperial College London, London, United Kingdom

**Keywords:** vaccine, vaccine development, infectious disease

## Abstract

The HIC-Vac network is a unique association of researchers focussed on the development and use of human infection challenge (HIC, otherwise known as controlled human infection models or CHIM) studies for vaccine and therapeutic development, particularly for pathogens of high global impact. The fifth annual meeting of the HIC-Vac network was held on 1–3 November 2023. The theme of the meeting was capacity-building in endemic settings particularly in low- and middle-income countries (LMIC), where pathogens cause the greatest morbidity and mortality. In this report we highlight the strengths and limitations of HIC and expansion of such studies into endemic settings, noting that immune responses and vaccine efficacy differ across diverse settings and populations. The consensus was that HIC studies must not be restricted to high income settings if they are to be relevant to LMIC populations. This report summarizes the work presented at the HIC-Vac annual meeting, highlighting current and future challenge models, challenge agent manufacture, public engagement, ethics, and industry perspectives.

## Graphical Abstract

**Graphical Abstract:**
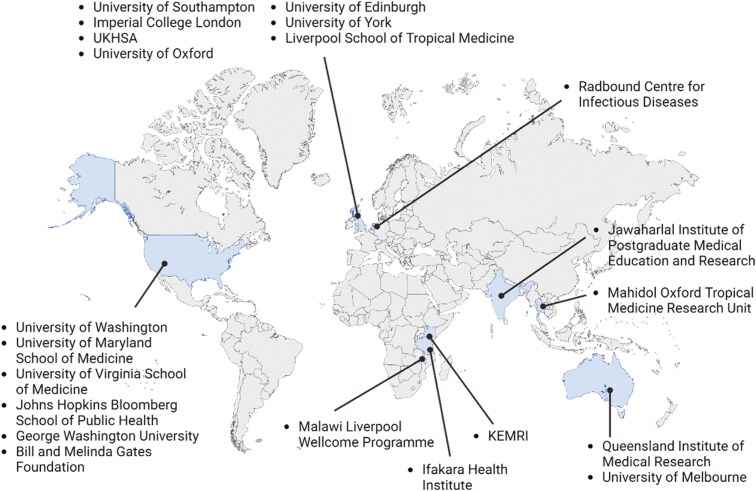

## Introduction

The HIC-Vac network was established in 2017 to advocate and support human infection challenge (HIC) studies (also referred to as controlled human infection models or CHIM, and Controlled Human Infection Studies or CHIS). The network is funded largely by the Medical Research Council (MRC, UK), the Biotechnology and Biological Sciences Research Council (BBSRC), and the Wellcome Trust, and more recently by the UK Research and Innovation (UKRI) International Science Partnerships Fund (ISPF). Daniella Ferreira (University of Oxford) replaced Andrew J Pollard as co-director, joining the Director, Peter Openshaw (Imperial College London), to lead the consortium.

**Figure 1.**
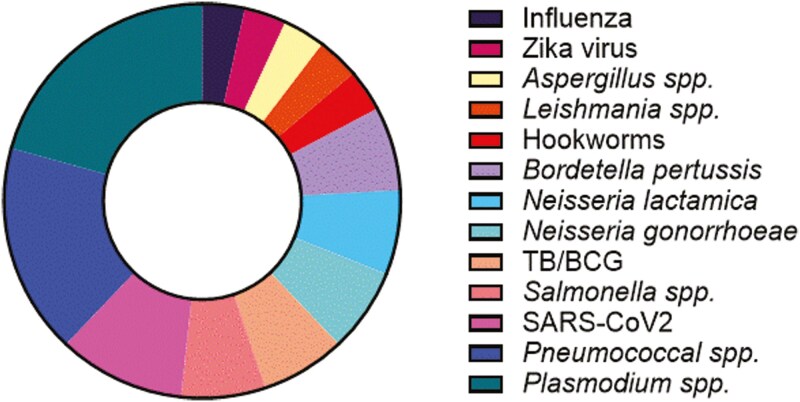
**Breakdown of the pathogens discussed in the context of established and potential human challenge models at the fifth annual meeting of the HIC-Vac network.** *Plasmodium spp., Pneumococcus spp.*, and SARS-CoV2 CHIMs made up almost half of the challenge studies discussed at the meeting, with the remaining presentations encompassing a broad range of viral, bacterial, and parasitic infections.

**Figure 2.**
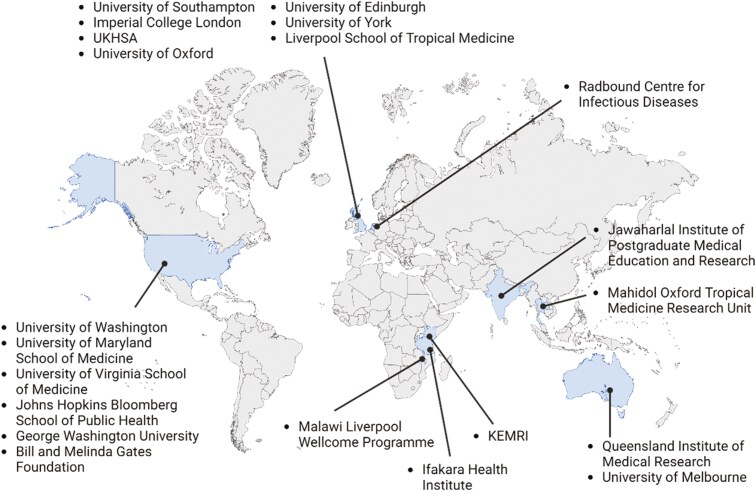
**Map of speakers and poster presenter location and affiliations.** Figure shows the diverse locations of presenters and affiliating institutions across high and LMIC.

Eighty-five delegates attended the 2023 fifth annual meeting of the HIC-Vac network in person, with another 28 online. The focus of the meeting was “Human challenge models for vaccine development – strengths, limitations, and expansion into endemic settings,” and sessions during the meeting covered a diverse mixture of viral, parasitic and bacterial challenge models (Fig. 1), both in progress and in development. There were 34 speakers and poster presenters, 6 of whom were recipients of pump-priming grants awarded by HIC-Vac to early-stage researchers. The speakers and poster presenters originated from a wide range of high and low/low-and-middle-income countries (LIC/LMIC), demonstrating the diversity and reach of the HIC-Vac network (Fig. 2).

A key objective of the HIC-Vac network is to support the development and use of vaccine-challenge studies. This unique type of study, in which participants receive a vaccine and are subsequently challenged with the pathogen of interest, has already de-risked several vaccine development pipelines (e.g. malaria, RSV), and promises to revolutionize the clinical trials landscape. Frederick Hayden from the University of Virginia School of Medicine and Keith Klugman from the Bill and Melinda Gates Foundation gave keynote presentations outlining lessons learned from challenge models on respiratory antivirals and vaccines, highlighting the global disease burden in children.

This report summarizes the current state of human challenge models presented at the 2023 HIC-Vac annual meeting and their use in vaccine development.

## Viral challenge models

### Update on the SARS-CoV-2 human challenge programme

The initial SARS-CoV-2 human challenge study was led by Imperial College London in 2021, the first and only example of a challenge study being set up and run during a pandemic [1]. The successful collaborative study, headed by Prof Christopher Chiu, was designed to optimize the model using the pre-Alpha strain in seronegative adults and answer fundamental questions about the virus such as the course of disease and host-virus interactions. The SARS-CoV-2 human challenge programme has longer term aims of providing a platform for the testing of new vaccines or antivirals with an emphasis on next generation transmission-reducing vaccines (e.g. mucosal vaccines) and validating correlates of protection. The more exploratory work generated from these exciting studies are providing rich and deep insight into early viral kinetics; the immune response in mild infection, especially pre-symptom onset that is often missed in natural infection studies; transmission [2]; host derived signatures for diagnostics [3]; and immune factors associated with viral control [4].

A novel and unexpected outcome of challenge with SARS-CoV-2 was the characterization of transient infection in which viral replication was detected by qPCR at single, non-consecutive timepoints. These participants did not develop sustained infection nor antibody responses and were asymptomatic. Detailed transcriptomic analysis revealed these participants did however mount a very early immune response associated with this beneficial outcome, particularly in the nasopharynx (the site of inoculation and viral replication) [5]. Data from this seronegative study and a subsequent study in seropositive adults [6] indicates pre-existing T cells as a major contributor to protection against sustained or transient infection. The programme is continuing with Delta and Omicron variant human challenge in seropositive individuals to model breakthrough infection and test mucosal vaccines.

### Zika virus

The outbreak of Zika virus, a mosquito-borne virus, in the Americas from 2015 to 2015 sparked a need for vaccines and therapeutics against this infection, which is associated with congenital malformations and Guillain-Barré syndrome. The waning of the outbreak meant that traditional large scale efficacy trials could not be carried out; therefore, a HIC model for Zika virus would not only contribute to the development of vaccines and therapeutics but also inform a better understanding of Zika virus infection [7]. A novel HIC study was set up by a team at the Johns Hopkins Bloomberg School of Public Health to evaluate two strains of Zika virus (ZIKV SJRP and ZIKV Nicaragua) in healthy; adult, non-pregnant women and men. Participants received virus or placebo and were monitored in an inpatient unit, rash and arthralgia were the most common symptoms, but both virus strains were well tolerated. Analysis of viral shedding kinetics and further studies, aiming to evaluate mosquito transmissibility, are ongoing.

## Bacterial challenge studies

### Bordetella pertussis

A resurgence of pertussis has been seen over recent years, despite high vaccine coverage. This resurgence is hypothesized to be due to the lack of protection against asymptomatic *Bordetella pertussis* (Bp) colonization and transmission afforded by the acellular pertussis vaccine [8, 9]. BPZE1 is a candidate live pertussis vaccine, genetically attenuated by the removal of dermonecrotic toxin and tracheal cytotoxin, and the inactivation of pertussis toxin [10]. When administered intranasally it induces transient nasopharyngeal colonization with the vaccine strain and induces local and systemic pertussis-specific humoral immune responses [11–13]. BPZE1 has previously been shown to have a favourable safety profile [11–13] and to protect against colonization at rechallenge with the vaccine strain [14].

The CHAMPION-1 study (University of Southampton and University of Oxford, UK; NCT05461131), was presented, although analysis is ongoing. This randomized, placebo-controlled phase 2b vaccine-challenge study was conducted to evaluate the impact of vaccination with BPZE1 on nasopharyngeal colonization with virulent Bp using the Bp controlled human infection model previously established at the University of Southampton [15, 16]. Participants were randomized 1:1 to vaccination with BPZE1 or placebo. 60–120 days after vaccination, participants in both arms of the study were challenged with 10^5^ CFU virulent Bp. Endpoints include colonization status and bacterial load as determined by culture of nasal wash samples following virulent challenge, and systemic and mucosal immunogenicity and safety parameters following both vaccination and challenge. The results of this study will be invaluable to the Bp vaccine development pipeline.

### Neisseria lactamica HIC in pregnancy

*Neisseria lactamica* is a harmless commensal that colonizes the upper respiratory tract of young children but is uncommon in infants and adults. In previous healthy adult HIC studies, nasal *N. lactamica* inoculation has been shown to be safe, achieves high colonization rates, and reduces *Neisseria meningitidis* colonization, potentially due to cross-reactive antibody and cellular immune responses [17, 18]. This model was therefore chosen for the world’s first human challenge study in pregnant women, which was set up and run at the University of Southampton [19]. It was hypothesized that nasal *N. lactamica* inoculation in pregnant women would result in infant colonization, providing a model for studying mother-to-infant commensal transmission and microbiome development. In fact, for the 21 inoculated women, no sustained mother-to-infant transmission was seen, despite evidence supporting mother-to-infant transmission of other commensals and broader microbiome sharing (publication in progress). These findings suggest respiratory commensal transmission between mother and infant is likely selective and microbe-specific. The study also demonstrated that HIC in pregnant women can be safe and acceptable [20], paving the way for future studies.

### BCG as a surrogate for tuberculosis in human challenge models

*Mycobacterium tuberculosis* (MTB) is the primary cause of infectious disease related mortality worldwide [21]. The BCG vaccine has been used to combat TB for decades, this vaccine however has limited impact on the transmission of MTB, and new vaccines and drug treatments are needed in the fight against TB mortality. The strengths and limitations of using BCG as a surrogate for MTB in human challenge models was discussed by E. Chandler Church (Fred Hutchinson Cancer Center). BCG challenge is administered intradermally and has been shown to be safe [22, 23], however, TB infection predominantly occurs in the lung thus posing a limitation to this model. Helen McShane (University of Oxford) and Keertan Dheda (LSHTM) have been studying alternative BCG administration routes by aerosol or bronchoscopy and data shows that this method is safe. Although currently this requires either inhalational administration, which results in an unpredictable bacterial load, or bronchoscopy, which presents procedural risks. Other hurdles for this model include the attenuated nature of BCG, which may not be induce the full magnitude or range of responses observed following infection with MTB, and the current lack of well characterized correlates of immune protection for either BCG and MTB.

A skin TICE® BCG challenge of healthy adults was set up to evaluate the bacterial burden in the skin using standard bacterial culture and viability PCR methods as well as extensive immunological evaluation of skin biopsy samples. This model was intended to validate BCG administration to the skin as a potential challenge model for antibacterial and host-directed therapies by studying bacterial burden before and after isoniazid and rifampicin administration, both of which are validated treatments for MTB. Although the isoniazid challenge failed to reduce bacterial burden, likely due to short administration time and higher activity against rapidly dividing bacteria, rifampicin showed an excellent reduction in bacterial burden. The results also demonstrated a complex cellular communication. This included muscle and epithelial cells interaction with immune cells, as well as communication between immune cell subtypes themselves. This study highlights one of the major differences between BCG and MTB, specifically the lack of rapid division within the site of infection. This ensures the safety of BCG, but does potentially limit its use in challenge studies combined with drug treatment intended to target rapidly dividing bacteria.

### Pneumococcal challenge studies (multiple sites)

Pneumococcal challenge studies are well established in the UK, with over 2500 participants challenged using 12 strains from 3 different serotypes and multiple vaccines tested to date [24, 25]. These models have also allowed researchers to investigate the responses of clinically vulnerable groups, such as asthmatics and older adults, as well as studying viral and bacterial co-infection. Daniela Ferreira, from the University of Oxford, presented the Pneumo2 study design; this is the largest human challenge study to date, with over 400 participants split into five study arms, aiming to study the effects of PCV13 and PPV vaccines against experimental pneumococcal colonization [26]. The early results of co-infection studies were also presented, demonstrating that rhinovirus, parainfluenza and RSV predispose individuals towards colonization with pneumococcal infections (Mitsi et al in preparation). These findings have led to the Respiratory syncytial virus and S. PnEumoniae Challenge Coinfection sTudy (RESPECCT) study design, which is currently recruiting participants to an inpatient CHIM, with the primary objective of determining whether RSV-A challenge increases the risk of secondary carriage by Spn6B (a serotype 6 pneumococcus).

Human challenge with serotype 3 (which, despite being included in both the available vaccines, causes approximately 10% of invasive Pneumococcal disease in the UK), is currently being set up at the Liverpool School of Tropical Medicine (LSTM). This serotype is of global importance, as it causes higher rates of sepsis and mortality, and a diminished vaccine effectiveness has been observed for this serotype. The study aims to optimize the model and assess local and systemic immune responses to serotype 3.

Andrea Collins also described LSTM’s current efforts to establish a large Human Challenge Facility, which will meet the UK’s un-met need for capacity building, providing a large, academic, non-NHS facility for in-patient CHIMs. This facility is also envisioned as a vital part of the UKs pandemic preparedness for ‘Disease X’.

Pneumococcal challenge studies, including feasibility, pilot and now a double-blind randomized controlled trial of PCV13 vs placebo in healthy adults have been successfully carried out in Malawi (Dula *et al*., 2023). The MARVELS consortium have also established the safety and acceptability of a pneumococcal challenge study in people living with HIV (PLHIV), as knowledge on the rate and density of pneumococcal carriage in PLHIV and correlates of protection in this group is lacking. Stephen Gordon of the Malawi Liverpool Wellcome Clinical Research Programme presented early results that showed acceptance was good in both PLHIV and HIV negative participants and there were no safety concerns. They also showed natural carriage in this group in Malawi was very common, with no invasive disease occurring in the observed cohort.

### Paratyphoid

*Salmonella* Paratyphi A is a major cause of enteric fever, responsible for 2.1 million cases every year and over 14000 deaths. The largest disease burden is in east Asia and sub-Saharan Africa, disproportionately affecting children and areas with poor sanitation and access to clean water. Current treatment with antibiotics is effective but difficulties in diagnosis and AMR lead to higher morbidity and mortality, therefore vaccine development is underway. A community acquired infection study to assess vaccine efficacy is considered difficult by the WHO, as the low attack rate means over 100,000 participants would need to be recruited [27]. A *Salmonella* Paratyphi A human challenge model has been established at the Oxford Vaccine Group and WHO has advised that this is a valuable method to assess efficacy of existing vaccines.

The vaccine-challenge study currently in progress to assess vaccine efficacy of a novel live attenuated, oral vaccine (CVD 1902 developed by University of Maryland, phase 1 study completed) against *Salmonella* Paratyphi A, was described by Naina McCann [28]. The *Salmonella* Paratyphi A challenge model, has previously been used to establish the challenge dose and to assess the impact of *Salmonella* Paratyphi A and *Salmonella* Typhi re-challenge [29, 30]. This current, multi-site study is the first to evaluate a Salmonella Paratyphi A vaccine in the model. The study will determine relative protection of 2 doses of CVD 1902 vs placebo and aims to assess the host response after vaccination and challenge in vaccinated vs placebo groups, investigating correlates of protection. Early results suggest the vaccine is safe and well-tolerated, with final results expected in early 2025.

### Non-typhoidal salmonella

NTS has a wide animal host range and can result in a spectrum of disease in humans. Gastroenteritis or diarrhoeal disease in humans is associated with low mortality, whereas invasive disease (iNTS), involving systemic infection, has a much higher fatality rate (estimated at 15–20%). iNTS mainly affects sub-Saharan African populations, especially children under 5 years old and adults with advancing HIV. Most iNTS cases are caused by *Salmonella Typhimurium*. There are no licenced vaccines for NTS yet, but there are several candidate vaccines in development (in both pre-clinical studies and phase I and II clinical trials).

Recently, a first-in-human non-Typhoidal *Salmonella* (NTS) controlled human infection study was set up at Imperial College London. Emma Smith presented the study design and aims [31]. This model may provide a future platform for testing vaccines for efficacy signals, alongside providing insights into mechanisms of disease and correlates of protection. The participants in the study are randomized to challenge with one of two strains (either 4/74, isolated in the UK and associated with diarrhoeal disease, or D23580, isolated in Malawi and associated with more frequent development of iNTS). The goal of this first study is to assess the safety of the model and identify a dose of each strain that results in an attack rate of approximately 60-75% in healthy adult volunteers, meeting primary endpoints indicative of systemic infection (fever or blood-borne bacteraemia). Further objectives of the study include comparing clinical and microbiological responses to the two different strains, and characterizing the host mucosal, cellular, and humoral immune response to challenge.

### Neisseria gonorrhoeae

*Neisseria gonorrhoeae* is a major global public health problem with increasing incidence of the disease world-wide in both high- and low-income settings [32]. Growing antimicrobial resistance and a broad spectrum of disease, with severe reproductive and neonatal health sequelae necessitates new treatments and prevention strategies. Vaccine development has been difficult due to the pathogen’s variable surface antigen expression, limited natural protection and dearth of known correlates of protection [33]. The modest vaccine efficacy of meningococcal B vaccines against *N gonorrhoeae* infection suggest a vaccine may be efficacious [34, 35]. A robust infection model, which will allow researchers to mimic human-pathogen interactions and trial next-generation vaccines and therapeutics, has been identified as a key objective in this field. Eloise Williams presented work on the CURE-NG project under development at the Peter Doherty Institute of Infection and Immunity at the University of Melbourne, which aims to develop the first human gonorrhoea oropharyngeal CHIM. The project is still in the early stages, with strain selection, clinical trial design and community engagement underway, but this CHIM may serve as a powerful platform for assessment of urgently-needed vaccines and therapeutics.

## Parasite challenge models

### Malaria human challenge studies across multiple sites

Controlled human malaria infection studies have been used widely as a tool to evaluate anti-malarial drug and vaccine candidates. Studies have been carried out in high and LMIC, including malaria endemic areas. Currently, the Mahidol Oxford Tropical Medicine Research Unit (MORU) and collaborators are conducting *Plasmodium vivax* controlled human infection studies in Thailand. However, CHIMs are relatively new, therefore researchers at MORU are conducting a social science study to evaluate the ethical considerations and acceptance of these studies in Thailand. Preliminary results were presented by Phaik Yeong Cheah and Bhensri Naemiratch, underlining the importance of considering the culture context within which CHIMs are taking place, as they found compensation was not the main incentive for volunteers, with a strong altruistic motive, compared to other settings previously studied.

Globally, progress towards malaria control has stalled in recent years, and resistance to artemisinin derivatives and their partner drugs continues to spread. Of particular concern is the recent emergence of artemisinin resistance in East Africa. There is a critical need for new drugs and antimalarial therapies, and malaria HIC studies can play a key role in expediating the drug development pipeline, whilst answering key questions about host-parasite interactions. Bridget Barber described an induced blood stage malaria challenge model, developed at the QIMR Berghofer Medical Research Institute, and its utility in clinical trials of several antimalarial drugs. She highlighted three recent studies at the unit; firstly a Phase 1b study evaluating blood stage activity of tafenoquine. Tafenoquine is a licenced antimalarial approved for chemoprophylaxis and for *P. vivax* radical cure (elimination of dormant hypnozoites), and the results presented demonstrate that tafenoquine also has activity in clearing asexual parasitemia and blocking transmission in volunteers infected with *Plasmodium falciparum* [36, 37]. Pyronaridine, a licenced drug used in combination with artesunate, has a high barrier to resistance, so there has been increased interest in this therapeutic agent in recent years. This drug was also tested in the induced blood stage malaria challenge model and results support the use of this drug in combination therapies [38]. The team are currently studying the use of Ruxolitinib, the Janus kinase (JAK1/2) inhibitor, at the time of anti-malarial treatment; this type I IFN signalling blocker has the potential to reduce the inflammatory response and disrupt the parasite-induced immune dysfunction [39]. This study found that Ruxolitinib was safe when administered alongside artemether-lumefantrine, and attenuated post-treatment inflammatory responses, demonstrating that these HICs can also help identify potential adjunctive treatments for severe malaria.

Controlled human malaria infection studies in non-endemic regions such as the UK, involve malaria naïve participants, with no prior exposure to malaria. Immunity to malaria in endemic areas develops with successive infections, resulting in tolerance to severe disease and eventually asymptomatic infections. In order to investigate these immune mechanisms, a *P. falciparum* malaria re-challenge (three times over 12 months) study was set up in Oxford by Angela Minassian and Simon Draper, in collaboration with Phil Spence and Wiebke Nahrendorf. The model revealed that although there was no change in clinical symptoms and inflammation upon successive second and third malaria infections, there was a lack of tissue damage in the second and third infections compared with the first. Assessment of the T cell response revealed that the excessive T cell activation seen in the first infection, was attenuated by the third, suggesting a controlled T cell response, preventing tissue damage that the authors hypothesize underpins immunity against severe malaria [40]. This type of study may become more important for comparing HIC and vaccine induced immune responses between non-endemic and endemic areas with high exposure levels.

### Leishmaniasis

Paul Kaye from the University of York presented the sand fly transmitted cutaneous leishmaniasis HIC developed to evaluate vaccine candidates. Leishmaniasis is a neglected tropical disease with high disease burden in Asia, sub-Saharan Africa and South America. The model was designed as a natural transmission HIC, with exposure of the human volunteer to *Leishmania major* through sand fly bite (sand flies being the natural vector for this infection). The method was shown to be safe and effective at inducing cutaneous leishmaniasis lesions and also addressed immunopathology and acceptability [41]. Future use of the HIC will include testing new vaccines or other preventative measures against the disease. Early results also showed how immunohistology and transcriptomic analysis can be utilized to measure immune responses in the lesions.

### Hookworm

Hookworm affects over 400 million people worldwide, with infection causing intestinal blood loss, iron deficiency anaemia and protein malnutrition. With the only currently approved treatment, albendazole administration, directed primarily at children, there is a clear clinical need for vaccines targeted at alleviating the substantial adult burden. There are several hookworm vaccines in development, however, transitioning to Phase 2 and 3 studies has proven difficult due to the recent SARS-CoV-2 pandemic and funding issues, therefore establishing a HIC would greatly benefit the drug development pipeline [42]. Feasibility and dose escalation studies were conducted at the George Washington University, in participants with no previous exposure history. Only mild to moderate adverse events were observed after application of hookworm larvae to the skin [43]. The early results of a randomized, placebo-controlled phase 2 trial, also conducted at the George Washington University, were presented by David Diemert. Hookworm-naïve adults were vaccinated three times with *Necator americanus* glutathione S-transferase-1 (Na-GST-1), a component of the hookworm blood digestion pathway [44], then challenged. Anti-Na-GST-1 IgG antibodies were induced and egg counts were found to be significantly lower in participants vaccinated with Na-GST-1 compared to placebo. The importance of transferring this model to an endemic region was discussed, as host exposure history may impact the immune response and therefore vaccine-induced protection compared with naïve, USA-based volunteers. Work is on-going to transfer the model to Brazil, however this has proven difficult, illustrating the challenges in obtaining ethical approval for CHIMs in different LMIC settings.

## Fungal challenge studies

### Pulmonary Aspergillosis

Aspergillosis is an opportunistic fungal infection, with high drug resistance, and limited treatment options for chronic pulmonary aspergillosis that occurs in populations with risk factors such as previous TB infection, chronic lung diseases, or immunological defects. There are new anti-fungal drugs in development and potential for a vaccine. This means a new challenge model would be invaluable in selecting which drugs and in what combinations would be optimal [45]. Darius Armstrong-James (Imperial College London) presented on the potential for these studies, the rational and difficulties posed. This type of study is in its infancy; therefore, comprehensive public involvement and engagement activity would be needed, to first establish the acceptability of intranasal *Aspergillus* inoculation. A pre-requisite to establishing this challenge model will involve addressing issues specific to this pathogen; notably that the population most at risk of infection are immunocompromised individuals, rather than the healthy participants needed to establish safety of the model, and that the infection is also predominantly within the lung, so infections limited to the nasal mucosa may not fully recapitulate natural disease.

## Other perspectives

### Public engagement and ethics

HIC-Vac recognizes that, despite the disproportionate burden of infectious disease affecting LMIC, a minority of CHIMs are carried out in these areas. Extensive discussions were therefore conducted surrounding the feasibility, acceptance and ethical considerations involved in establishing HIC in endemic settings, especially in LMIC. Several discussions, already mentioned throughout the meeting, touched on this in a variety of LMIC, culminating in a talk by Venkateswaran Ramanathan from the Jawaharlal Institute in India, who expanded on the experience of embarking on controlled human infection studies in an endemic area, highlighting the initial steps and considerations for countries embarking upon HIC studies. Perspectives from different populations were considered in the context of establishing a Typhoid CHIM in India; ethics committee members from institutions across India and the general public from Bangalore and Mugalur [46]. While CHIMs have yet to be attempted in India, there is an acceptance of the need for CHIMs amongst the general public, although this is accompanied by concerns regarding the safety and regulation of these studies, highlighting the need for sustained public engagement regarding risk mitigation.

This was addressed in a panel discussion involving Helen Ward (Imperial College London), Kondwani Jambo (Malawi Liverpool Wellcome Programme), Peter Openshaw (Imperial College London), and Melissa Kapulu (KEMRI-Wellcome Trust Research Programme). The discussion was chaired by Tom Darton and touched on such topics as; the importance of involving the public in designing trials, empowering and giving autonomy to volunteers, and the need to contextualize risk and communicate this effectively to volunteers. An interesting point was raised regarding informed consent about risk considering the range of pathogens HIC-Vac members research, and how this differs by pathogen and endemic/non-endemic setting.

The power of HIC-Vac members to spread awareness and advocacy about CHIMs was demonstrated by the premiere of the short film ‘Emerging from the pandemic: The power of challenge trials’.

### Industry perspective

There is increasing interest in the use of CHIMs to support decision making regarding investment in large, time-consuming field trials and efficacy studies for vaccine licensure within the biopharmaceutical industry. Stephen Lockhart (Imperial College London) presented misconceptions about CHIMs he has encountered in internal company discussions, which preceded a panel discussion on the use of challenge studies in product development. Perspectives concerning the applicability of CHIMs to vulnerable populations versus healthy adults and defining correlates of protection were discussed. It was agreed that working with industry, collaboratively, can answer specific questions on immune biomarkers, such as upper or lower respiratory tract mucosal immunity correlates of protection, that are currently not feasible in larger scale field trials. Knowing the immune response desired, vaccine candidate selection, dose or regimen selection can also be honed by CHIM studies, aiding decision making. An important opposing perspective was also considered, as growing reliance upon CHIM studies have led to a vaccine candidate being dropped or led to decisions delaying vaccine development.

### GMP challenge agent manufacture

The production of challenge agents for use in HIC was discussed in a session delivered by the Smart Practices Group, a consortium of international experts providing regulatory advice regarding the fundamental principles of selection, characterization, manufacture, quality control, and storage of challenge agents. The agent is usually selected for manufacture in advance of the study design and protocol finalization, and this influences the manufacturing requirements and the design of the clinical study (i.e. strain selection, dose-escalation elements, and route of administration). Early engagement with regulatory bodies is recommended, as there may be requirements to involve advisory groups, agencies regulating medicines, GMO, and importation authorities. The agent is extensively characterized and put though a QC process to check identity by sequencing, as well as purity, potency, and stability. There was an in-depth discussion on the benefits and drawbacks surrounding implementation of good manufacturing practice (GMP), as the requirements differ between countries and depends upon the phase of the study. If a HIC study directly contributes to the licencing of a vaccine, it was suggested that GMP should be considered, however in other circumstances, it is not always possible to use GMP facilities, and emphasis should instead be on strain selection, infectivity characterization and purity.

## Conclusions from the meeting

Far-ranging discussions on current and newly proposed HIC studies highlighted the global impact of the consortium, especially in enabling projects in resource-poor settings where the need for new vaccines is high. It was agreed that studies of pathogens in endemic settings must be used to address outstanding questions, discovering correlates of protection in relevant populations and fast-tracking scoping studies of vaccine immunogenicity and efficacy. Poster presentations were especially valuable for early career researchers and recipients or pump-priming grants to discuss future collaborations and grant applications.

The vaccine field is moving fast, galvanized by the success of COVID-19 vaccines and a deeper understanding of protective immunity. There is greater understanding of the interplay between viruses and bacteria, with a growing realization that infections build upon each other; a rich history of innate and acquired immunity in mucosal surfaces contributes to a landscape in which pathogenic or protective immunity develops to new infectious challenges. Human challenge studies have played a transformative role in these achievements and the HIC-Vac programme can be proud of the part it has played, and will continue to play, in this expanding field of knowledge that underpins vaccinology.

## Data Availability

Data sharing is not applicable to this article, as no new data were created or analysed in this study.
